# Trace Metal Pollution in Topsoil Surrounding the Xiangtan Manganese Mine Area (South-Central China): Source Identification, Spatial Distribution and Assessment of Potential Ecological Risks

**DOI:** 10.3390/ijerph15112412

**Published:** 2018-10-31

**Authors:** Feng Jiang, Bozhi Ren, Andrew S. Hursthouse, Yingying Zhou

**Affiliations:** 1 Hunan Provincial Key Laboratory of Shale Gas Resource Exploitation, Xiangtan 411201, China; jiang6feng@163.com (F.J.); andrew.hursthouse@uws.ac.uk (A.S.H.); yingyzhouq@163.com (Y.Z.); 2 School of Civil Engineering, Hunan University of Science and Technology, Xiangtan 411201, China; 3 Computing Engineering & Physical Sciences, University of the West of Scotland, Paisley PA1 2BE, UK

**Keywords:** manganese mining, topsoil, trace metals, source identification, spatial distribution, potential ecological risk assessment

## Abstract

In this study, we identified the sources of trace metals, investigated their spatial distribution in topsoil and assessed their potential ecological risk in the area surrounding a typical manganese mining area in Xiangtan, Hunan Province, China. The concentrations of Mn, Cu, Pb, Zn, Cd, Ni, Cr and Hg in the topsoil of the study area were measured. Except for Cr and Hg, all trace metals exceeded the corresponding soil background values for Hunan Province. The spatial variation in trace metals was visualized by GIS, and the results show that trace metals in topsoil are enriched mainly around mines and smelters. Two groups of trace metals were identified using the spatial distribution, trend analysis, Pearson’s correlation and principal component analysis: Mn, Cu, Pb, Zn, Cd and Ni can be attributed to industrial and mining activities, whereas Cr and Hg are of natural origin. The results also revealed the extent of the influence of secondary processes such as the prevailing wind direction, erosion of mine tailings and rainwater runoff play significant roles in the wider dispersal and transfer of trace metals. In addition, the environmental risk of metal pollution was evaluated by applying the geoaccumulation index and potential ecological risk index (PERI) to the study area. The accumulated PERI for metals of concern is at highest risk level in the main manganese mine area. This decreases to a moderate risk around the manganese mine area, highlighting locations for further risk management concern. Furthermore, nearly 80% of the potential ecological risk was from Cd across the study area.

## 1. Introduction

Mining activities are regarded as a primary source of trace metals in the environment [1]. A large volume of mine waste that contains various trace metals is produced as a result of mining and smelting, causing ecological damage and serious trace metal pollution problems in mining areas [2,3]. The main impacts of trace metal pollution are areas of wasteland and metal-rich runoff from waste-rock heaps and the subsequent contamination of terrestrial and aquatic environments. On the one hand, trace metal toxicity is persistent and accumulative through the food chain and can inhibit the growth and reproduction of plants and microbes [4]. For instance, excessive intake of copper (Cu) can cause Wilson, Parkinson’s, Alzheimer’s, and prion diseases [5], and inorganic arsenic can cause neurological disorders, skin diseases and cancer [6]. Cadmium (Cd) can cause lung cancer, prostatic hyperplasia, and renal dysfunction [7]. Lead (Pb) can cause damage to bones, blood circulation and endocrine and immune systems [8,9]. In addition, trace metal pollution in soil can also lead to direct contamination of surface and groundwater and through physical transport can accumulate in soil and enter the river system through surface runoff and leachates, leading to secondary contamination of the surface water and groundwater. The growth and reproduction of aquatic plants, diatoms and benthic invertebrate communities in river basins are inhibited by excessive concentrations of trace metals [10]. Therefore, trace metal pollution in soils is a serious global problem and has received extensive attention from the academic community.

There are numerous studies in the literature on the ecological risk of trace metals in rivers, lakes, sediments, industrial areas, and agricultural areas [11,12,13,14]. However, correlative analyses of trace metal sources, distribution characteristics and ecological risk assessments in manganese mine areas are rare. The spatial distribution of trace metals in topsoil is largely influenced by natural sources (ore weathering and leaching) and human activities (mining, production and domestic sources) [15]. Correlation analyses are used to establish correlations among various types of trace metals [16]. Principal component analysis (PCA) has been proven to be an effective tool that can be used to identify potential sources of trace metals [17]. Trend analysis diagrams better reflect the trends in sample data in study areas [18]. In addition, the spatial variation and distribution of trace metals in soil have been visualized using geostatistics to identify the source [19]. The geoaccumulation index (i.e., I_geo_) is widely used to assess the environmental significance of enrichment of individual elements. The potential ecological risk index (i.e., PERI) is a relatively rapid and simple method for assessing the potential ecological risk of trace metals in soils to the environment [1]. In addition, kriging interpolation has been used to visualize the spatial distribution of the pollution degree (I_geo_) and ecological risk (PERI) of trace metals in soil to more intuitively analyze the data. This can aid in prioritization of risk management strategies and evaluate the magnitude of intervention needed.

Following previous studies, a typical manganese mine and its surrounding area were taken as an example to study the sources and spatial distribution of trace metal pollution in soil and determine the ecological risks of soil trace metals. The effects of natural factors on the diffusion and transportation of trace metals were predicted. It was found that large amounts of manganese and associated trace metals enter the mining area and the surrounding soil from surface runoff, wind-borne transportation and atmospheric sedimentation, which causes serious trace metal pollution in the mining area. An ecological risk assessment was performed in the study area to determine whether trace metals in the topsoil need ecological remediation. This study can provide a valuable reference for the evaluation of trace metals in topsoil in manganese mining areas and other nonferrous metal mining areas that have trace metal pollution issues, and these results can also provide an important theoretical basis for the control and remediation of soil pollution.

## 2. Materials and Methods

### 2.1. Study Area

**Figure 1:**
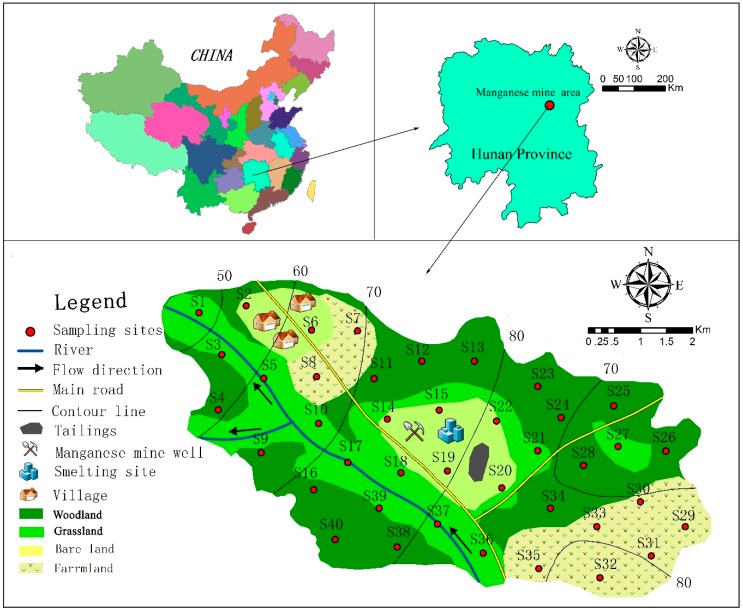
Simplified map of the study area and sampling locations.

The study area is located in the Xiangtan manganese mining area (111°58′–113°05′ E, 27°21′–28°05′ N), which covers an area of 20 km^2^ in the east central region of Hunan Province (Figure 1). The Xiangtan manganese mine is an important source of raw manganese in China with a long history of mining development [20]. The mine is located in a humid climate region within the middle subtropical monsoon climatic zone that has distinct seasons and abundant sunshine [21]. Spring and autumn are short, while winter and summer are long. Spring lasts from March through April, summer lasts from May to September, autumn occurs in October, and winter lasts from November to February. The annual mean temperature and rainfall are approximately 17.4 °C and 1300 mm, respectively [21]. Rainfall varies substantially with the seasons, and the prevailing wind direction is to the northwest. The main industries are mining and metal smelting, which are closely related to trace metal pollution in the manganese ore area of Xiangtan city [20]. Several different landscapes surround the mining area (woodland, grassland, farmland, villages, rivers, and mining and smelting areas). Main plants belong to herbaceous species, such as Lavandulaefolia DC., Juncus effusus, Miscanthus floridulus, Erigeron annuns, etc. [21].

### 2.2. Sample Collection and Treatment

#### 2.2.1. Sample Collection

Forty topsoil samples (0–20 cm) were collected from evenly distributed sampling sites in the study area, and the sampling interval was moderate (Figure 1). The longitude and latitude coordinates of the sampling points were recorded by GPS, and the sampling number and time were also recorded at the same time. A total of 0.2 kg soil was collected from the sampling site and another 4 soil samples were collected at locations 5 m from north, east, south and west directions. A composite sample of 1.0 kg was prepared from the 5 soil samples. Together, these subsamples formed a 1.0 kg sample that was stored in a polyethylene bag and transported to the laboratory. Samples were air-dried indoors and then disaggregated and ground into a powder using a porcelain mortar and pestle. The samples were sifted through a 0.9 mm nylon screen to remove impurities such as stones, rough materials and leaves.

#### 2.2.2. Analysis and Quality Control

Metal concentrations were determined following standard procedures [22]. A total of 0.25 g of dry sample was weighed in a Teflon digestion vessel with 12 mL aqua regia. Subsequently, the samples were digested on a graphitic panel heater for 2 h. Once the digestion was completed, the extracts were cooled, filtered and diluted to 50 mL with distilled water to separate any insoluble silicate particles. The total concentrations of Mn, Cu, Pb, Zn, Cd, Ni and Cr in the extracts were analyzed using inductively coupled plasma-atomic emission spectrometry (ICP-AES, JY38S, Jobin Yvon, Longumeau, France). Hg was analyzed by atomic fluorescence spectrometry (AFS-230E, AFS, Beijing, China). Analytical data quality was verified using quality assurance and quality control (QA/QC) that included the analysis of reagent blanks, duplicate samples and standard reference materials (GSS-1, GSS-8, GSS-10 and GSS-11) for each batch of samples. The error of the replicated samples analysis was less than 10%, and the error between the measured values and the certified values was less than 5%.

### 2.3. Analytical Methods

#### 2.3.1. Multivariate Statistical Analysis

SPSS 22 (IBM, Armonk, NY, USA) was used to analyze the statistical eigenvalues of the calculated data, and a Pearson correlation analysis was used to describe the correlation between trace metals, which provided effective information to interpret the trace metal sources and pathways in the environment [23]. PCA was used to transform multiple variable indicators into a few comprehensive indicators and further assist with identifying the trace metal sources in soil from the manganese mining area [24]. A curve regression analysis was used to determine the relationship between the concentration of trace metals at the sampling sites and the distance from the central area of the manganese mine [25].

#### 2.3.2. Geostatistical Analysis

In ArcGIS 10.3 (Esri, Redlands, CA, USA), the ordinary kriging method was employed to estimate the spatial data of the non-sampled sites [26]. Using ArcGIS mapping technology, the spatial distribution, degree of pollution and ecological risk of trace metals in the study area were represented in graphical form. A trend analysis diagram was made using ArcGIS to better reflect the trends throughout the sample data [27].

### 2.4. Assessment Methods

#### 2.4.1. Geoaccumulation Index (I_geo_)

The geoaccumulation index (I_geo_) was proposed by Muller [28] from the Institute of Sediment Research, Heidelberg University, Germany. This index is important for evaluating the degree of trace metal pollution in sediments by using the trace metal contents of the particles to reflect the pollution level. The equation for the calculation is:

(1) $$I_{\mathrm{geo}}{=\log}_{2}\frac{\mathrm{Cn}}{K*\mathrm{BEn}},$$

where Cn is the concentration of the metal examined in the samples, and BEn is the geochemical background concentration of that metal. K is a constant that is used to modify the variation in background values caused by differences in rock composition between different places (K is generally assigned a value of 1.5). The geoaccumulation index consists of seven grades or classes, indicating the degree of pollution from uncontaminated to serious pollution (Table 1).

During the calculation of the geoaccumulation index, the average geochemical background value of a trace metal element in the global shale is usually chosen as the background concentration value. However, it is difficult to represent the background values of sediments formed by different sedimentation processes by choosing ordinary shale as the geochemical background value; thus, the trace metal pollution information obtained does not accurately reflect the pollution situation [29]. Based on the above considerations, when using the I_geo_ and PERI (see Section 2.4.2) to evaluate the trace metal pollution and ecological risk in sediments, geochemical background values as close as possible to the geochemical characteristics and environmental characteristics of the sediments in the area should be selected. This procedure can ensure that the calculated I_geo_ and PERI values are in accordance with the actual situation of the area, and thus ensure the accuracy of the trace metal pollution analysis. The background values in this study are taken from the Chinese National Environmental Monitoring Center’s (CNEMC) “The Background Values of Chinese Soils” [30], which contains background values of different soil layers in various provinces of China. In this study, the soil background values of the A-layer (0–20 cm) in Hunan Province are selected. The sampling points were located in undisturbed soil that was not in areas of known contamination. The results obtained by using global ordinary shale and terrestrial soil values of China as the geochemical background values of I_geo_ are basically consistent [30].

#### 2.4.2. Potential Ecological Risk Index (PERI)

The PERI, which was proposed by Hakanson [31], considers the toxicology of trace metals for evaluating the potential ecological risk caused by the overall levels of contamination in surface sediments. Ecological risk levels are shown in Table 2 and are calculated using the following formula:

(2) $$\mathrm{RI}=\sum E_{r}^{i}=\sum T_{r}^{i}\left(\frac{C_{s}^{i}}{C_{n}^{i}}\right),$$

where RI is the sum of individual potential ecological risks from all trace metals, $E_{r}^{i}$ is the potential ecological risk index of a single element, $T_{r}^{i}$ is the toxic-response factor for a given trace metal, $C_{s}^{i}$ is the present concentration of trace metals in topsoil, and $C_{n}^{i}$ are the background concentrations of the Hunan Province pre-industrial era. The toxic-response factors for Mn, Cu, Pb, Zn, Cd, Ni, Cr, and Hg are 1, 5, 5, 1, 30, 5, 2, and 40, respectively [32,33]. In the present study, the background concentrations of trace metals in soil from Hunan Province were taken as reference values (see above (1)) [30].

## 3. Results and Discussion

### 3.1. Physio-Chemical Characteristics

The concentrations of Mn, Cu, Pb, Zn, Cd, Ni, Cr and Hg are 4.19, 2.00, 8.36, 2.05, 12.86, 1.60, 0.27 and 0.69 times greater than the background concentrations in soil from Hunan Province, respectively (Table 3). According to the variation coefficients, the coefficient of variation (CV) values for Mn, Cu, Pb, Zn, Cd and Ni are 112%, 52%, 128%, 82%, 159% and 49%, respectively. The considerable variability that exists in the element data indicates that the spatial distribution of these elements is not homogeneous [34]. In addition, the high variability may be related to natural variations and external factors [35]. Trace metal concentrations that far exceed the background concentration values and the high variability (35% < CV) of Mn, Cu, Pb, Zn, Cd, and Ni suggest that their concentration distributions may be determined by factors including natural variation and human activities, such as the emission of pollutants from industrial enterprises, wind erosion of tailings, and mining and smelting activities [25]. The CV values of Cr and Hg (32% and 34%, respectively) show a moderate degree of spatial variation (15% < CV ≤ 35%) with a relatively homogeneous spatial distribution. However, the average concentrations of Cr and Hg in the topsoil were lower than the background values in Hunan Province. Thus, the moderate variability in Cr and Hg may be controlled by natural factors.

### 3.2. Interelement Relationships and Possible Sources

A Pearson correlation analysis was used to calculate a correlation coefficient matrix to study correlations between trace metal concentrations (Table 4). A significant positive correlation between trace metals indicates a common origin and similar pathways [25,36]. The correlations among Mn, Cu, Pb, Zn, Cd and Ni are significant (0.01 significance level), and the correlation coefficients between them are greater than 0.6 (Table 5). This result means that these six elements have some homologous features, which suggests that they may share a common source. The results also show that there is no significant correlation (0.01 significance level) between Cr and Hg and other trace metals in the study area, indicating that these two elements possibly originated from various sources.

Through PCA (Table 5), two principal components are obtained with eigenvalues greater than 1 that explain 77.9% of the variation in the dataset. According to the component matrix (Table 3), the first principal component (PC1) includes Mn, Cu, Pb, Zn, Cd, and Ni and contributes 63.4% of the cumulative variance. These six trace metals are present at relatively high concentrations and high CV values, which is indicative of disturbance by external factors (i.e., mine drainage, tailings, mining and smelting activities) [25]. Mining operations and smelter emissions are considered the main factors that affect the accumulation of trace metals in soil [37]. The relatively lower CV values compared to those of the other six element groups indicated the relatively homogeneous distributions of Cr and Hg in the study area. Moreover, the mean concentrations of Cr and Hg were below the background values in soil of Hunan Province, indicating that they were undisturbed by the external environment. The second principal component (PC2) includes Cr and Hg (low concentration), which mainly originated from natural sources.

**Figure 2:**
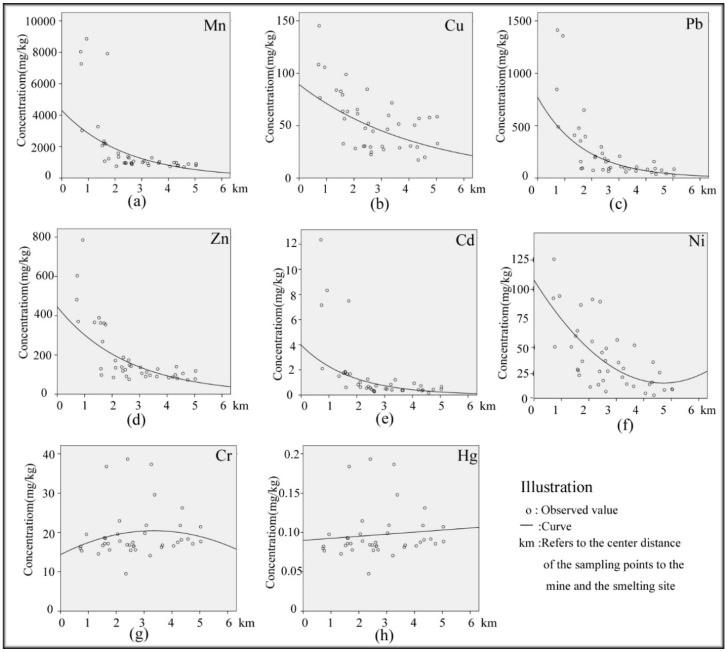
Scatter diagrams of trace metal concentrations as a function of distance: (**a**) Mn; (**b**) Cu; (**c**) Pb; (**d**) Zn; (**e**) Cd; (**f**) Ni; (**g**) Cr; (**h**) Hg.

To further identify the influence of manganese mining and smelting on trace metal sources in the study area, scatter diagrams of the trends in the trace metal concentration variations were constructed as a function of increasing distance from the smelting site using curvilinear regression analysis (Figure 2). Slightly elevated concentrations of Cr were found within 3 km of the smelter, and concentrations gradually decreased with increasing distance from the smelting site. With increasing distance, the concentration of Hg gradually increased. The variation in the trends of the Cr and Hg concentrations may be caused by natural factors in the study area. Li et al. [25] also came to a conclusion similar to that reached above. As shown in Figure 2, the trends in the trace metal (Mn, Cu, Pb, Zn, Cd and Ni) concentration variations show a clear decline with increasing distance from the location of manganese mining and smelting. In conclusion, manganese mining and processing have a measurable influence on the trace metal (Mn, Cu, Pb, Zn, Cd and Ni) concentrations in the study area. This result is probably caused by the diffusion of the waste gas from the smelter and wind erosion of mine tailings [38,39,40,41]. Second, with the relatively higher elevations (S14, S15, S19–S22) of mines and tailings, trace metals diffused through rainwater runoff, and a similar result was reported in many articles [27,42,43,44,45].

### 3.3. Spatial Distribution of Trace Metals

#### 3.3.1. Trend Analysis

**Figure 3:**
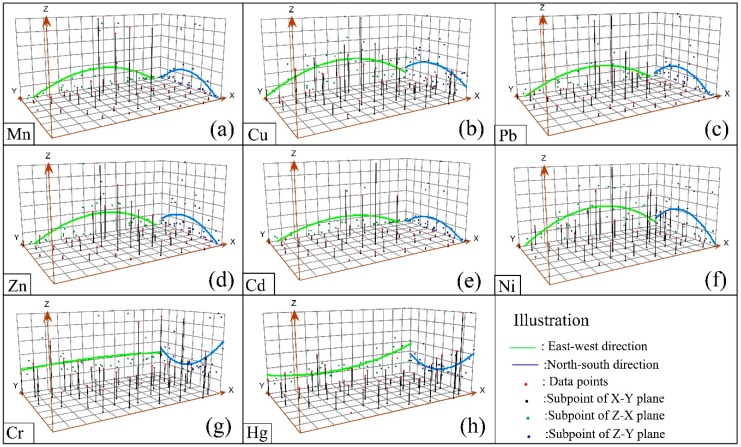
Results of trend analyses for the geographical distribution of trace metal concentrations: (**a**) Mn; (**b**) Cu; (**c**) Pb; (**d**) Zn; (**e**) Cd; (**f**) Ni; (**g**) Cr; (**h**) Hg.

The height of each vertical line in the trend analysis (Figure 3) represents a sample concentration value. By projecting these sample values in the north–south direction (the *zy* plane in Figure 3) and the east–west direction (the *zx* plane in Figure 3), a curve simulating the concentration trend in the east–west or north–south directions can be obtained [27]. The Mn, Cu, Pb, Zn, Cd, and Ni concentrations in the north–south and east–west projections show an inverted “U” shape (Figure 3). Combined with the topography and an industrial layout analysis of the study area, this trend may be caused by the fact that the mines and smelters are located in the center of the study area. The concentrations of six trace metals (Mn, Cu, Pb, Zn, Cd, and Ni) increase toward the center of the study area as approached from both north to south and east to west. The trend analysis of the sample data for Cr and Hg in the study area form a “U” shape from north to south (Figure 3), indicating that there is a two-dimensional trend function in the north–south direction. The Cr concentration is high on both sides and low in the middle of the study area; however, the maximum Cr concentration measured at the sampling sites is lower than the background value, indicating that there is no Cr pollution in the study area. The Hg concentration is close to the background value, indicating that there is practically no Hg contamination in the study area. The measured Cr and Hg concentrations are projected onto straight curves in the east–west direction (Figure 3), and a straight line close to the level is obtained. This trend suggests that the Cr and Hg concentrations in the study area are dominated by natural sources [27].

#### 3.3.2. Interpretation of the Spatial Distribution of Trace Metals

**Figure 4:**
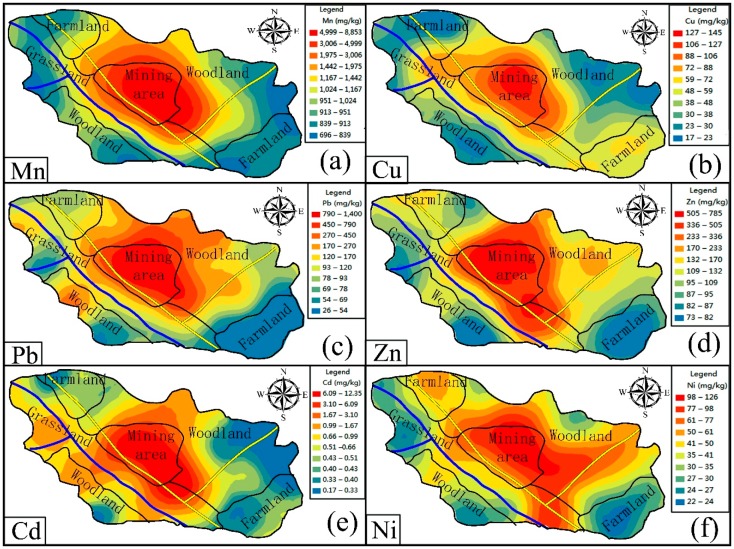
Concentration distribution maps of trace metals (Mn, Cu, Pb, Zn, Cd, and Ni) in soil in the study area: (**a**) Mn; (**b**) Cu; (**c**) Pb; (**d**) Zn; (**e**) Cd; (**f**) Ni.

The kriging interpolation method was performed on all samples to obtain visual information on the spatial distribution of trace metals (Figure 4). The spatial distribution of Cr and Hg concentrations shows that their distribution trend is not clearly related to the mining and smelting of manganese, which is consistent with the results of the multivariate statistical analyses and suggests that their distribution may be the result of natural factors. Figure 4 shows that elevated concentrations of Mn, Cu, Pb, Zn, Cd and Ni are centered around the mine, smelter and tailings.

From the spatial distribution maps of Mn, Cu, Pb, Zn, Cd and Ni (Figure 4), the following three observations are summarized. First, the distribution trends of Mn, Cu, Pb, Zn, Cd and Ni are similar, and this fact indicates that the pollutants are released from a common source. Second, the concentrations of trace metals (Mn, Cu, Pb, Zn, Cd, Ni) are the lowest in the east and highest in the mine, smelter and tailings areas. The spatial distribution maps (Figure 4) are consistent with the results of the regression analysis (Figure 2) and trend analysis (Figure 3). Third, the mine and smelter are located in the center of the study area, and there is a phenomenon of high concentration “enrichment” on the spatial distribution map, which leads to the conclusion that trace metal pollution is mainly sourced from the mining and smelting of manganese. According to the environment of the area surrounding the manganese mining area, waste gas from the smelter is discharged directly into the atmosphere without treatment. In the absence of other sources of pollution, the degree of Mn, Cu, Pb, Zn, Cd and Ni pollution in the main wind direction (northwesterly) is slightly higher than that in other directions (Figure 4). This result is because the smoke that contains trace metals is diffused by wind and enters the topsoil through dry and wet deposition from the atmosphere, which results in differences in the distribution patterns of metals in the soil [31,46]. The prevailing wind direction is consistent with the distribution trends of trace metals in soil (Figure 4). Zn and Ni concentrations are also high in the southeast, which may be related to the wear of automobile tires [47] and the automobile exhaust emissions from urban and road traffic [48,49,50] rather than from the influence of the manganese mine. This result clearly shows that Zn and Ni have sources other than mining activities. Figure 4 shows that at the same distance from the mining site, the levels of copper and cadmium in farmland and rivers are higher than those in woodlands and grasslands, indicating that fertilizers and pesticides have an effect on trace metals [51]. Therefore, we infer that the pollution of Cd and Cu is also affected by local agricultural activities such as the application of fertilizers and pesticides. In addition, as seen from Figure 4, trace metals are enriched in the river. In addition, tailings are located in areas of high topography, and rainwater runoff is another mechanism for the transport of trace metals. Vegetation coverage at the sampling sites (Figure 1) could also influence the distribution of trace metals in soils, as the trace metal concentrations are relatively low in woodland soil [25]. Generally, the low trace metal concentrations in vegetated areas are easily affected by both natural factors and human activities [52].

### 3.4. Degree of Pollution and Ecological Risk Assessment

#### 3.4.1. Degree of Pollution

**Figure 5:**
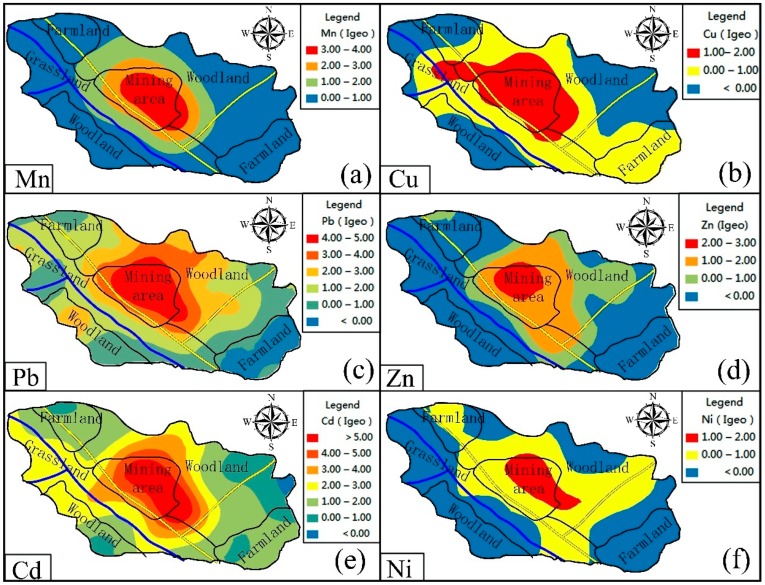
Geoaccumulation index (I_geo_) maps of Mn, Cu, Pb, Zn, Cd, and Ni in topsoil in the study area: (**a**) Mn; (**b**) Cu; (**c**) Pb; (**d**) Zn; (**e**) Cd; (**f**) Ni.

Topsoils were found to be relatively uncontaminated by Cr and Hg because of their low I_geo_ values in the study area. Although the geoaccumulation index of Ni is close to zero, the high variability indicates that there is slight local pollution in the study area, which may be caused by human activities or natural variations. The I_geo_ values of Cu and Zn in topsoil correspond to uncontaminated to moderately contaminated levels, the I_geo_ values of Mn and Pb in topsoil correspond to moderately contaminated levels, and the I_geo_ values of Cd in the topsoil correspond to moderately to heavily contaminated levels. The degree of trace metal pollution in descending order is Cd > Pb > Mn > Cu > Zn > Ni. The I_geo_ values for Mn, Cu, Pb, Zn, Cd, and Ni have a large CV values (Table 6), indicating a large variation in the degree of pollution in the study area, which could have been caused by external factors [35]. In addition, interpolation with the kriging method was performed on all I_geo_ values of the six trace metals to determine the distribution of the degree of pollution (Figure 5), which further shows that smelting and mining activities are the main sources of trace metals in the environment. However, some trace metals were moderately contaminated along roads, farmlands and rivers far from the mining sites (Figure 5). This result suggests that secondary sources of trace metals in the study area exist. One interesting finding is that trace metal pollution is highest in the mining sites at higher altitude (highlands), and lower pollution levels found in the area surrounding low altitude sites (lowlands) (Figure 5). In addition, the degree of trace metal pollution in the direction of prevailing wind is higher than that in the other directions, and the concentrations of trace metals in low-lying water bodies such as rivers are higher than those in other places (except for the mining area). After natural weathering or leaching, trace metals in ore may diffuse and transfer throughout the mining area by means of wind, river transport, and surface runoff [41]. A similar result was reported in a number of study areas [21,32,47,48,49,50]. According to the geoaccumulation index (I_geo_) maps of trace metals (Figure 5), Ni is moderately polluted along the highway, and Cu and Cd are also moderately polluted in the locations where the farmland is located. This pattern may be because vehicle emissions are also a source of Ni pollution, and Cu and Cd distribution are also affected by agricultural activities [17,53,54].

#### 3.4.2. Ecological Risk Assessment

**Figure 6:**
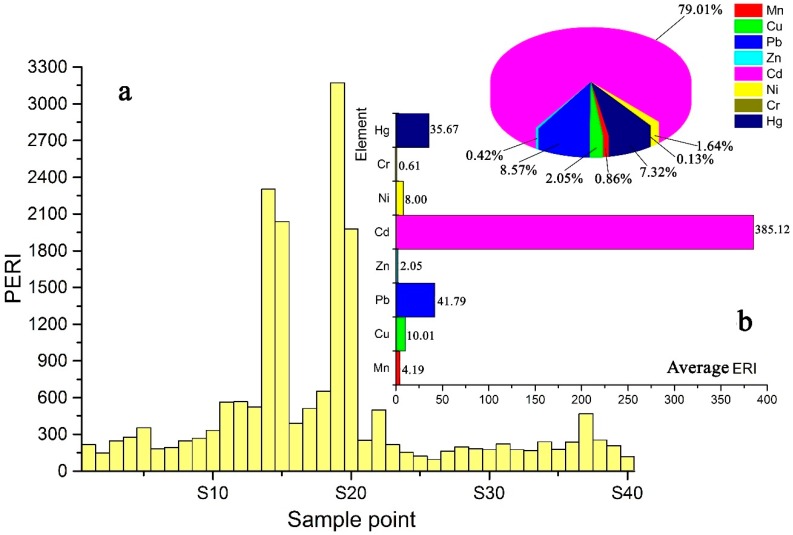
Cummulative PERI data for individual samples (**a**) and $E_{r}^{i}$ average contribution from individual elements (**b**).

I_geo_ values can reflect the degree of pollution for a single trace metal but not the integrated influence of multiple metal pollutants. Therefore, it is necessary to use the PERI to further evaluate the ecological risk of trace metals in topsoil. The $E_{r}^{i}$ values and PERI values of eight trace metals are illustrated in Figure 6, and the order of the average potential ecological risks of single metals is Cd > Pb > Hg > Cu > Ni > Mn > Zn > Cr. In this order, Hg is the 3rd (Figure 6b), and, based on the previous studies (spatial distribution and I_geo_), Hg showed low concentration and uncontamination. This is because the toxicity response factor of Hg is very high compared with Mn, Cu, Pb, Zn, Ni, Cr (the toxic-response factors for Mn, Cu, Pb, Zn, Cd, Ni, Cr, and Hg are 1, 5, 5, 1, 30, 5, 2, and 40, respectively). The ecological risk for Cd is serious in the study area, with 45% of the sampling sites at a moderate ecological risk level (in woodland, grassland and farmland), 20% at a high ecological risk level (in mining area), 27.5% at a very high ecological risk level (at almost all mining sites), and only 7.5% of the sampling sites at a low ecological risk level (in woodland). The ecological risk levels for Cd are related not only to smelting and mining activities but also to the higher toxic-response coefficient for Cd. Pb is at a moderate ecological risk level near the mine and smelter (S14, S15). The average ecological risk factors for Hg, Cu, Ni, Mn, Zn and Cr are generally at low levels (Figure 6).

**Figure 7:**
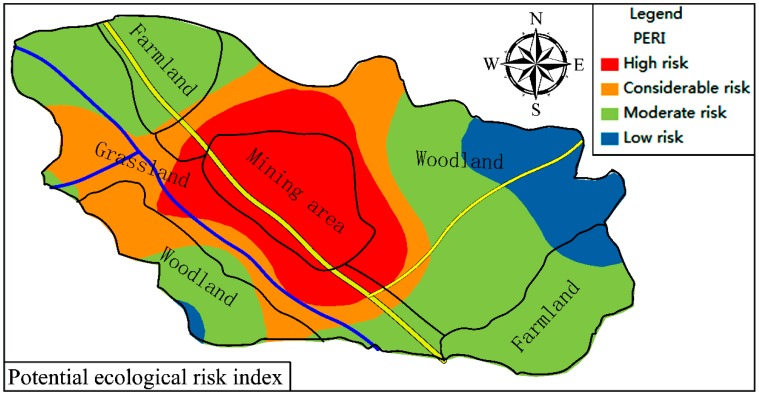
The spatial distribution of cumulative PERI from metals in topsoil in the study area.

The range of the PERI for trace metals in topsoil is 97.13–3171.91 (Figure 6), and Cd contributes on average 79.01% to these values. A total of 55% of the sites are at moderate ecological risk (in woodland and farmland), 22.5% are at considerable ecological risk (in almost all grassland areas), 12.5% are at high ecological risk (in the mining area), and only 10% are at low ecological risk (in woodland) (Figure 7). Therefore, the ecological risk of trace metals in topsoil is relatively high in the study area. Cd contributes the most significant potential ecological risk (Figure 6). The contributions of Pb and Hg are 8.57% and 7.32%, respectively. The total contribution of Cu, Ni, Mn, Zn, and Cr is only 5.10% (Figure 6). The potential ecological risk index for Cd at different sampling sites is in good agreement with the corresponding PERI values. Therefore, the PERI of each sampling site is related to the potential ecological risk of Cd, and Cd pollution in topsoil in the study area should be monitored closely and prioritized in future assessment and management strategies.

The distribution of the PERI for trace metals in topsoil in the study area is shown in Figure 7, which highlights that the majority of the PERI values are above a moderate ecological risk level in the study area. In addition, the potential ecological risk values of trace metals gradually decrease as a function of distance from the center of the mine area, which also indicates that trace metal pollution in the study area is mainly sourced from mining and smelting and that the pollution is serious and widespread.

## 4. Conclusions

The source, spatial distribution and ecological risk of trace metals (Mn, Cu, Pb, Zn, Cd, Ni, Cr, Hg) in the topsoil of the Xiangtan manganese mining area and the surrounding area were analyzed. This study provides a valuable reference for other mining areas that have similar trace metal soil pollution levels and can assist with the control and remediation of trace metal pollution. The conclusions are as follows:

(1) Mining is the main source of pollution, but secondary sources of pollution from traffic and agriculture are also of concern. Pearson’s correlation and PCA were used to identify two groups of trace metals. The results of the PCA showed that the contribution rates of the first two components were 63.397% and 14.498%. Mn, Cu, Pb, Zn, Cd and Ni are affected by human activities such as mining and smelting, while Cr and Hg are of natural origin.

(2) Trace metal dust in the waste gas of the smelter appears to be dispersed locally by wind and enters the soil through atmospheric deposition. Trace metals contained in tailings after wind erosion enters the soil and rivers through rainfall and surface runoff both in dissolved and suspended materials. All of these transport mechanisms contribute to the spatial distribution of trace metals observed.

(3) According to the I_geo_ and PERI results, there is no/low Cr and Hg pollution in the study area; there is slight Cu, Zn and Ni pollution, moderate Mn and Pb pollution, and heavy Cd pollution. The PERI of trace metals in topsoil in the study area is mostly above the moderate risk level, and the contribution of Cd to the PERI in the study area is nearly 80%; therefore, Cd is considered the main pollutant and the area deserves increased attention.

(4) Management of the topsoil contamination requires different approaches. The primary waste from mining needs to be remediated as a priority. The use of fertilizers and pesticides in agriculture should be avoided as far as possible to isolate secondary sources of pollution. Secondary emission sources to air—industrial and vehicle should be treated and controlled at the source. This study has identified the local scale of the priority areas that need to be controlled. The management needs vary from location to location and need to be incorporated in a site wide strategy.

## Figures and Tables

**Table 1:** Geoaccumulation index and contamination levels.

Classes	I_geo_	Pollution Level
0	I_geo_ ≤ 0	Practically uncontaminated
1	0 < I_geo_ ≤ 1	Uncontaminated to moderately contaminated
2	1 < I_geo_ ≤ 2	Moderately contaminated
3	2 < I_geo_ ≤ 3	Moderately to heavily contaminated
4	3 < I_geo_ ≤ 4	Heavily contaminated
5	4 < I_geo_ ≤ 5	Heavily to extremely contaminated
6	I_geo_ > 5	Extremely contaminated

**Table 2:** Indices and grades of potential ecological risk.

Index	Grades of Ecological Risk Pollution				
	Low Risk	Moderate Risk	Considerable Risk	High Risk	Very High Risk
$E_{r}^{i}$	$E_{r}^{i}$ < 40	40 ≤ $E_{r}^{i}$ < 80	80 ≤ $E_{r}^{i}$ < 160	160 ≤ $E_{r}^{i}$ < 320	$E_{r}^{i}$ ≥ 320
RI	RI < 150	150 ≤ RI < 300	300 ≤ RI < 600	RI ≥ 600	

**Table 3:** Descriptive statistics for trace metal concentrations and basic parameters in soils sampled in this study (mg/kg).

Element	Max	Min	Mean ± SD	CV (%)	Background Values ^b^
**Mn**	8853.12	696.40	1922.41 ± 2149.82	112	459
**Cu**	145.26	17.14	54.64 ± 28.42	52	27.3
**Pb**	1411.20	26.13	248.25 ± 317.96	128	29.7
**Zn**	785.27	73.16	194.69 ± 160.27	82	94.4
**Cd**	12.35	0.17	1.62 ± 2.57	159	0.126
**Ni**	126.29	22.41	51.02 ± 25.13	49	31.9
**Cr**	38.64	9.50	19.42 ± 6.20	32	71.4
**Hg**	0.20	0.04	0.08 ± 0.027	34	0.116

^a^ Min = minimum; Max = maximum; CV = coefficient of variation; SD = standard deviation; ^b^ Based on values in The Background Values of Chinese Soils. Beijing, China. Chinese Environmental Science Press: Beijing, China, 1990.

**Table 4:** Pearson correlation coefficients for trace metals in topsoil.

	Mn	Cu	Pb	Zn	Cd	Ni	Cr	Hg
**Mn**	1							
**Cu**	0.798 **	1						
**Pb**	0.906 **	0.791 **	1					
**Zn**	0.869 **	0.774 **	0.935 **	1				
**Cd**	0.954 **	0.738 **	0.844 **	0.797 **	1			
**Ni**	0.732 **	0.672 **	0.767 **	0.755 **	0.676 **	1		
**Cr**	−0.125	−0.03	−0.221	−0.095	−0.115	0.016	1	
**Hg**	−0.184	0.116	−0.242	−0.259	−0.189	−0.241	0.113	1

** Indicates that the correlation is significant at the 0.01 level.

**Table 5:** Component contribution and individual trace metal contribution to data variance.

Component	Initial Eigenvalues			Element	Component	
	Total	% of Variance	Cumulative %		PC1	PC2
1	5.072	63.397	63.397	Mn	0.962	−0.024
2	1.160	14.498	77.895	Cu	0.851	0.357
3	0.944	11.799	89.694	Pb	0.964	−0.080
4	0.352	4.404	94.098	Zn	0.940	−0.025
5	0.234	2.293	97.020	Cd	0.917	0.011
6	0.156	1.956	98.976	Ni	0.835	0.038
7	0.049	0.611	99.597	Cr	−0.134	0.645
8	0.033	0.413	100.000	Hg	−0.232	0.779

**Table 6:** Geoaccumulation index statistics table.

Element	Min	Max	Mean ± SD	CV (%)
**Mn**	0.02	3.68	1.00 ± 1.02	102%
**Cu**	−1.26	1.83	0.23 ± 0.75	326%
**Pb**	−0.77	4.99	1.74 ± 1.38	79%
**Zn**	−0.96	2.46	0.12 ± 0.92	767%
**Cd**	−0.15	6.03	2.20 ± 1.42	64.5%
**Ni**	−1.09	1.40	−0.06 ± 0.66	−1100%
**Cr**	−3.32	−1.30	−2.35 ± 0.39	−17%
**Hg**	−1.75	0.57	−0.81 ± 0.41	−51%
